# Clinical and laboratory characterization of cutaneous leishmaniasis in Chinese migrant workers returned from Iraq

**DOI:** 10.1371/journal.pntd.0012006

**Published:** 2024-03-04

**Authors:** Kuo Bi, Xiaoli Li, Rui Zhang, Xiaoyan Zheng, Fei Wang, Yang Zou, Lei Wang

**Affiliations:** 1 Department of Pathology, Beijing Friendship Hospital, Capital Medical University, Beijing, PR China; 2 Beijing Institute of Tropical Medicine, Beijing Friendship Hospital, Capital Medical University, Beijing, PR China; 3 Beijing Key Laboratory for Research on Prevention and Treatment of Tropical Diseases, Beijing, PR China; 4 Clinical Laboratory Center, Beijing Friendship Hospital, Capital Medical University, Beijing, PR China; Charité University Medicine Berlin, GERMANY

## Abstract

**Background:**

Imported cutaneous leishmaniasis (CL) is a growing problem with increasing global travel to endemic areas. Returned travelers with CL are easy to be misdiagnosed and mistreated due to the lack of awareness for the disease to the physicians in non-endemic region that may lead to unfavorable outcome. Our study intends to summarize the characteristics of *Leishmania* infection imported from Iraq, so as to help Chinese physicians diagnose and treat the disease. **All CL patients were treated with intralesional injection of antimony.**

**Methods:**

The definitive diagnosis of CL is based on the parasite identification by microscopic examination directly on lesion smear or parasite culture, PCR amplification of *Leishmania*-specific internal transcribed spacer 1 (ITS-1). The phylogenetic analysis, the immunopathological examination **and** the cytokine detection were proceeded after the diagnosis.

**Results:**

We have identified 25 CL cases in migrant Chinese workers returned from Iraq for the first time with *L*. *major* as the major species of infected *Leishmania* parasite. Clinical features of the Iraq-imported CL include the history of skin exposure to sandflies bite and the lesions mostly on the exposed limbs. More ulcerative wet lesion was observed than nodular dry lesion. PCR is not only used to detect *Leishmania* parasite with high sensitivity, but also to identify the species of infected parasite through sequencing the amplified *Leishmania*-specific ITS-1 gene. The phylogenetic analysis based on the amplified ITS-1 sequences revealed that the infected *Leishmania* was closed related to the species and strains endemic in Iraq. The immunopathological examination revealed the T-cell filtrated cellular immune response with less B cells and NK cells involved. The cytokine profile measured in the skin lesion also confirmed the Th1 cellular response with higher expression levels of IFN-γ, IL-6 and IL-8. The skin lesions in CL patients were healed after being treated locally with antimony.

**Conclusions:**

The clinical and parasitological features of these Chinese CL cases imported from Iraq provide useful information for the diagnosis and treatment of CL that is not commonly seen in Chinese local population.

## Author summary

Cutaneous leishmaniasis (CL) is a worldwide disease caused by *Leishmania* and it is a threat to public health in developing countries. Because of the non-specific skin lesion of CL and very low number of CL cases found only in Northwest China desert, it is easy to be misdiagnosed as other skin issues, especially in non-endemic region of China. As economics grows, an increasing number of Chinese travelers to endemic countries and returned with CL. While physicians in China are unfamiliar with the various cutaneous manifestation of CL, the misdiagnosis may lead to inappropriate treatment and unnecessary delay of healing. Our study summarized the clinical and parasitological features of 25 Chinese CL cases imported from Iraq for the first time, which include epidemiology, the diagnostic methods and species identification of infected *Leishmania spp*.. All these features of imported CL cases could help Chinese physicians with the diagnosis and treatment of CL in China.

## 1. Introduction

Leishmaniasis is a vector-borne disease caused by intracellular kinetoplastid protozoa *Leishmania spp*., transmitted by the bite of infected female sandflies. More than 12 million people are infected worldwide with estimated 0.7–1.0 million new cases in almost 100 endemic countries annually [1,2]. The disease ranges from asymptomatic infection, self-curing cutaneous lesions to life-threatening visceral leishmaniasis.

Visceral leishmaniasis (VL), also called kala-azar, is the most severe form of leishmaniasis caused by the infection of *L*. *donovani* in Asia and Africa and *L*. *infantum* in the Americas [3], Mediterranean and Middle East region [4]. Cutaneous leishmaniasis (CL) is usually limited to an ulcer or lesion in skin, caused by the infection of *L*. *major*, *L*. *tropica*, *L*. *infantum*, *L*. *aethiopica*, and *L*. *donovani* in old world (mostly in middle east or Sudan) or *L*. *braziliensis* and *L*. *mexicana* in new world (mostly in Brazil, Peru and Colombia) [5]. Even though most of CL is self-healing over 3 months to 5 years depending on the infected species, up to 10% of them progress to more severe manifestations called mucocutaneous leishmaniasis [1,6,7]

In China, VL was one of the most serious parasitic diseases and seriously endemic in 16 provinces in 1950s transmitted by the bite of sandfly carrying promastigote of *L*. *donovani* [8]. It has been effectively controlled after decades’ effort of case treatment and vector control in the most area of China [8,9], currently with only around 250–300 new cases reported annually in the northwest desert areas based on WHO Global Health Observatory [10] and other studies [11,12]. CL is barely seen in China, with sporadic cases reported in the Gobi desert area of west China, mainly caused by *L*. *infantum*, the species believed to be circulated in local animals [11,13–15]. Currently, the cutaneous leishmaniasis is mainly pandemic in the regions of central Asia and Middle East countries including Iraq, caused mostly by the infections of *L*. *major* and *L*. *turanica* and *L*. *tropica* [16]. Iraq is a country with high prevalence of CL, caused mostly by the infection of *L*. *major* [17].

As China economy has rapidly and steadily grown over the past decades, China increased its economic activities in the developing countries, especially after the launch of the Belt and Road Initiative (BRI) policy. China has sent a lot of migrant workers overseas, especially to the countries of Africa and Middle East where the CL is endemic. Only in Iraq, China invested more than US$ 10.5 billion and nearly 20 000 Chinese engineers and workers were dispatched there in 2021 [18]. As these migrant workers returned to China, they brought back a lot of imported CL cases from those endemic countries with high prevalence of CL, such as North Africa, Middle East and Central Asia [19–22].

Although CL has already been recognized as one of ten leading skin diseases in the migrating people returning from endemic countries [23], physicians in China are still unfamiliar with the wide variety of cutaneous manifestations of imported CL. In this study, we investigated 72 suspected CL cases returned from Iraq and found 33 cases with confirmed CL. In this study, we reported and analyzed the major clinical manifestation, immunological characteristics and the diagnostic criteria of imported CL, to facilitate the better diagnosis and treatment of cutaneous leishmaniasis for Chinese physicians and parasitologists. In addition, DNA sequencing and phylogenetic analysis have been conducted on the infected species to better understand the evolutionary relationship among closely related strain in Iraq and neighbor countries.

## 2. Materials and Methods

### 2.1. Ethics statement

This project was approved by the Ethics Committee of Beijing Friendship Hospital (Beijing, China) with approval number 2022-P2-294. Written informed consents were obtained from all participants involved in this study and the identity of each participant was protected and never be released.

### 2.2. Patients and diagnostic criteria

The patients returned from Iraq with suspected CL were admitted to the Beijing Friendship Hospital, Capital Medical University from August 2015 to May 2022.

The diagnosis of cutaneous leishmaniasis was made based on the recommendations of Clinical Expert Consensus on Diagnosis and Treatment of Leishmaniasis in China [24], the Clinical Practice Guidelines by the Infectious Diseases Society of America (IDSA) and the American Society of Tropical Medicine and Hygiene (ASTMH) [25]. The confirmed case of CL should meet following criteria: (1) living history in *Leishmania* endemic area, (2) cutaneous lesions; (3) laboratory parasite confirmation including amastigote identification in smears from the lesions margin with Giemsa staining, or histopathological examination of skin biopsy with H&E and/or periodic acid-schiff staining, or promastigote in the culture from scrapings in Novy-MacNeal-Nicolle medium, or positive molecular amplification of *Leishmania* internal transcribed spacer 1 (ITS-1) DNA in skin scrape specimen using quantitative real-time PCR assay as previously described [26]. Due to the poor antibody response in cutaneous leishmaniasis, serological assay is not recommended for the diagnosis of CL and has not done in this study [25, 27].

### 2.3. Immunohistochemistry of dermal lesion tissue

Immunohistochemical tests were performed on lesion skin tissue collected from patients. Briefly, the tissue was formalin fixed and paraffin-embedded. The skin tissue sections were recognized by primary antibodies against CD4, CD8, CD19 and CD56 (Becton Dickinson, San Jose, California), respectively, and visualized with the Labeled Streptavidin–Biotin (LSAB) system (Dako, Carpinteria, USA) according to the manufacturer’s instructions [28, 29]. The positive staining area were measured by Image J system (version 1.48) compared to the total area of lesion under the same view field. The results were shown as the average of three different field ± SD for each lesion sample, and the final positive staining areas were shown from total 15 cases as (positive area/total lesion area of skin tissue) × 100%.

### 2.4. Cytokine expression in lesion tissue

Skin lesion biopsy samples were collected from each CL patient and snap frozen in liquid nitrogen. Total RNA was extracted from these lesion samples and reverse-transcribed to cDNA using SuperScript Reverse Transcriptase kit (Invitrogen, Carlsbad, California). The primers and probes for detecting each cytokine cDNA were listed in Table 1. The housekeeper β-actin gene was added as control. Real-time quantitative PCR (RT-qPCR) was performed using Applied Biosystems 7500 Fast Real-Time PCR System. Comparative C_T_ (ΔΔCT) method was used to calculate differences in cytokine gene expression value compared to housekeeping β-actin gene as control.

**Table 1:** Oligonucleotide sequences of primer pairs used for real-time PCR.

Cytokines gene	Forward	Reverse
IFN-γ	GCTGCTGATGGGAGGAGATG	TGTCTGGCCTGCTGTTAAAGC
IL-4	GAACAGCCTCACAGAGCAGAAGAC	TGTCGAGCCGTTTCAGGAATC
IL-6	CACATGAACTGTGTTTGCCGCCTGGT	GCAGCCTTGTCAGCACACCTGGGAGCTGTAGA
IL-8	ATGACTTCCAAGCTGGCCGTGGCT	TCTCAGCCCTCTTCAAAAACTTCTC
IL-10	TCTATTCTAAGGCTGGCCACACT	CAATTGAAAGGACACCATAGCAAA
β-actin	ATGGATGACGATATCGCT	ATGAGGTAGTCTGTCAGGT

### 2.5. DNA sequencing and phylogenetic analysis

To determine the species of infected *Leishmania*, the internal transcribed spacer I (ITS-1) gene of *Leishmania* was amplified from patient lesion samples using specific primers: forward 5′-CTG GAT CAT TTT CCG ATG-3′ and reverse 5′-TGA TAC CAC TTA TCG CAC TT-3′, then sequenced. The obtained ITS-1 sequences were aligned using CLUSTALW. The phylogenetic tree of isolated species was constructed based on the Neighbor-joining (NJ) methods.

### 2.6. Treatment of CL

The CL cases with definitive diagnosis were treated with intralesional injections of 1–3 mL sodium stibogluconate (SSG) depended on the size of lesion, daily for 6 consecutive days [30].

### 2.7. Data acquirement

All data, including clinical and laboratory information from each patient, were input into a database for analysis.

### 2.8. Statistics

Continuous variables are presented as median [interquartile range] and categorical variables as numbers (frequencies). Categorical variables were compared using chi-square test or Fisher’s exact test as appropriate. Continuous variables were compared using Student’s t-test or Wilcoxon-Mann-Whitney test as appropriate. A two-tailed *p*-value <0.05 was considered statistically significant. Statistical analyses were performed using R software 3.1 version (R Development Core Team, 2008) using GMRC Shiny Stat application developed by CHU de Strasbourg (2017).

## 3. Results

### 3.1. Epidemiological and demographic features of the patients returned from Iraq

**Fig 1:**
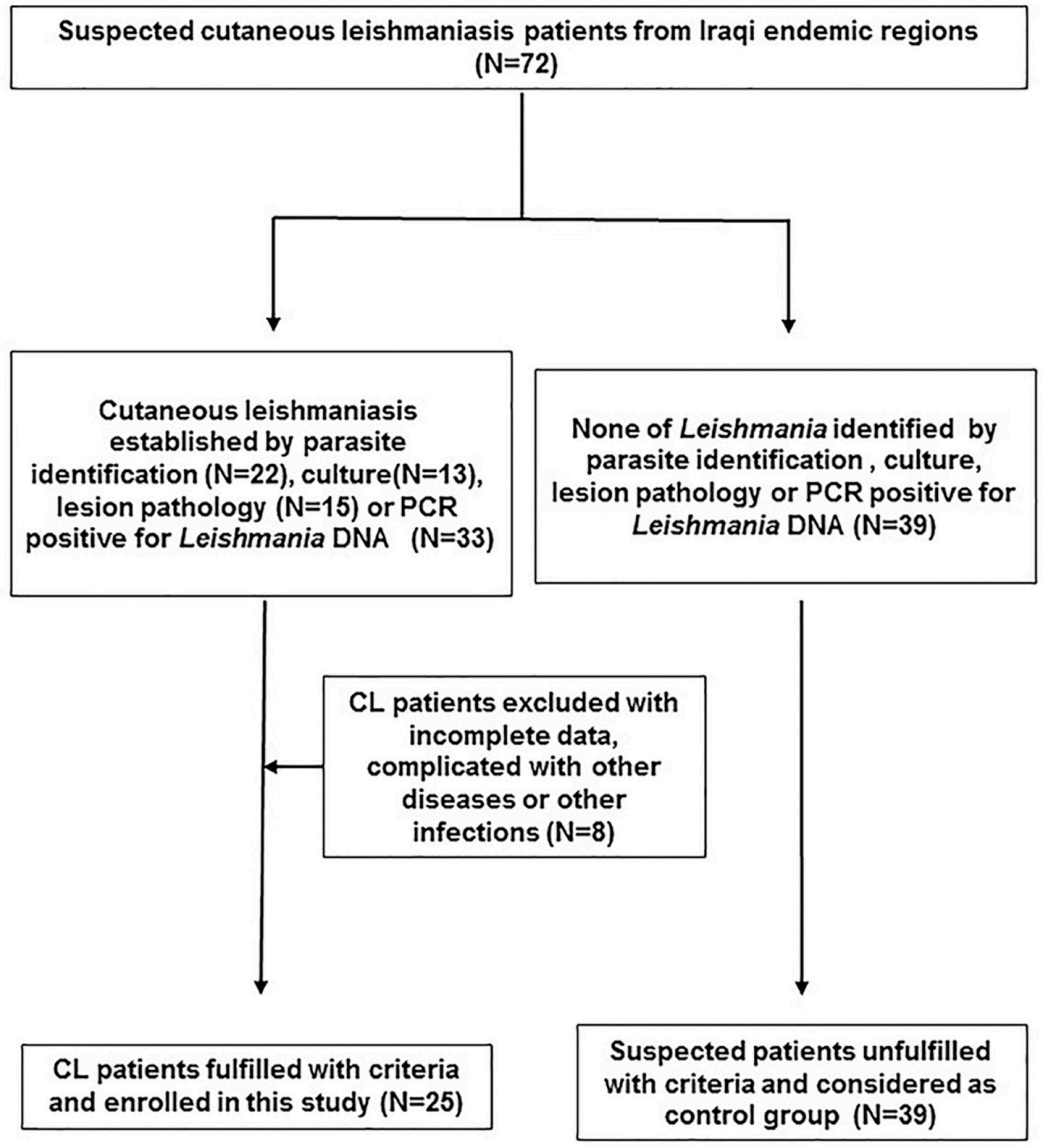
Flow chart for the patient selection of CL group and control group.

A total of 72 patients returned from Iraq with suspected cutaneous leishmaniasis were hospitalized from August 2015 to May 2022. All patients received diagnostic tests including the parasite examination (microscopy or PCR or parasite culture), and 33 cases were diagnosed as current CL based on the diagnostic criteria set at Materials & Methods. Among them, 8 cases were dropped from the study due to the conflict of availability or other physical disorders. Thus, the left 25 cases were considered as confirmed cutaneous leishmaniasis and enrolled in this study. The left 39 cases who were unfilled with the diagnostic criteria were used as control group (Fig 1).

Among the 25 confirmed CL cases, all patients were male, more than a half were construction workers (15/25, 60%) followed by workers with transportation (5/25), engineer (3/25) and administrator (2/25). Higher rate of construction workers in CL group than control group reflects these outdoor workers have higher chance to be bitten by sandflies carrying parasite although most of them claimed to have used insect repellent during their outdoor work (16/25, 64%). In addition, most of the confirmed cases (21/25, 84%) had chance to contact with dogs, which is significantly higher than people who had not been infected (10/39, 25.6%). The epidemiological and demographic features were summarized in the Table 2 as followed.

**Table 2:** Demographic and potential risk factors of the returned infected cases versus uninfected populations.

Parameters		Number of infected cases (%)	Number of uninfected populations (%)
Age			
	21–30	2 (8)	5 (12.8)
	31–40	14 (56)	22 (56.6)
	41–50	9 (36)	12 (30.6)
Gender			
	Male	25	39
	Female	0	0
Occupation in Iraq			
	Engineer	3 (12)	7 (17.9)
	Construction worker	15 (60)	15 (38.5)*
	Transportation	5 (20)	16 (41)*
	Administrators	2 (8)	1 (2.6)
Exposure to dogs			
	Yes	21(84)	10 (25.6)*
	No	4 (16)	29 (74.4)*
Use of DEET insect repellents			
	Yes	16 (64)	31 (79.5)*
	No	9 (36)	8 (20.5)*

*indicates *P* value less than 0.05 between two groups.

### 3.2. Geographic distribution of CL cases infected in Iraq

**Fig 2:**
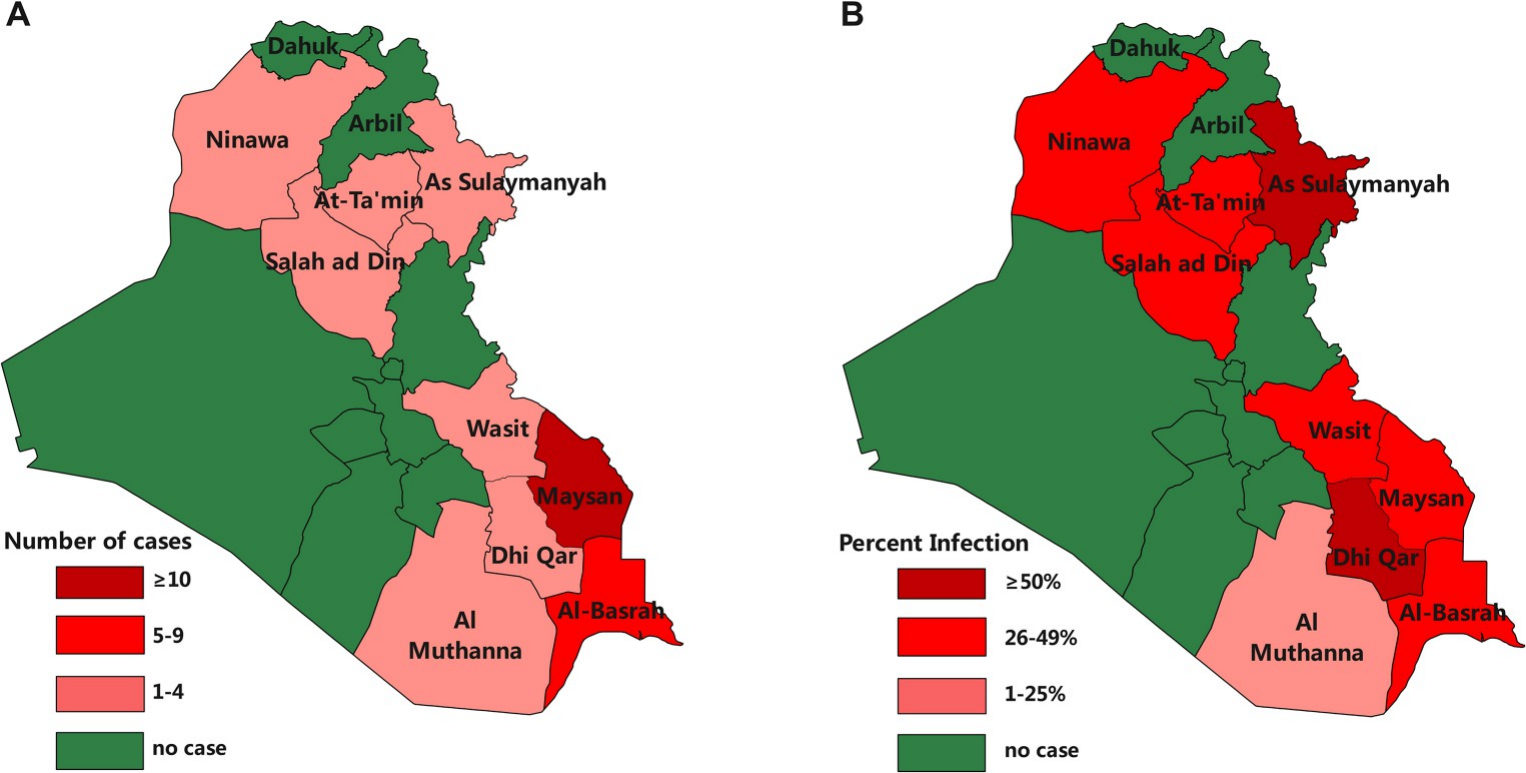
Province-based geographical distribution of Chinese cutaneous leishmaniasis patients in Iraq. A. Geographic distributions of Chinese CL cases. **B.** Geographic distributions of local CL prevalence.

Most of infected cases came from two provinces in Iraq including Maysan and Al-Basrah (20/25, 90%) where have high prevalence of CL in local population. The geographic distribution of infected Chinese cases is correlated with the prevalence of CL in local population even though the highest prevalence rate occurs in Sulaymaniyah and DhiQar. The result presented that Chinese patients of cutaneous leishmaniasis in this study were mainly infected in the Northern and Eastern areas of Iraq (Fig 2).

### 3.3. Clinical characterization and diagnosis of the patients with CL returned from Iraq

**Fig 3:**
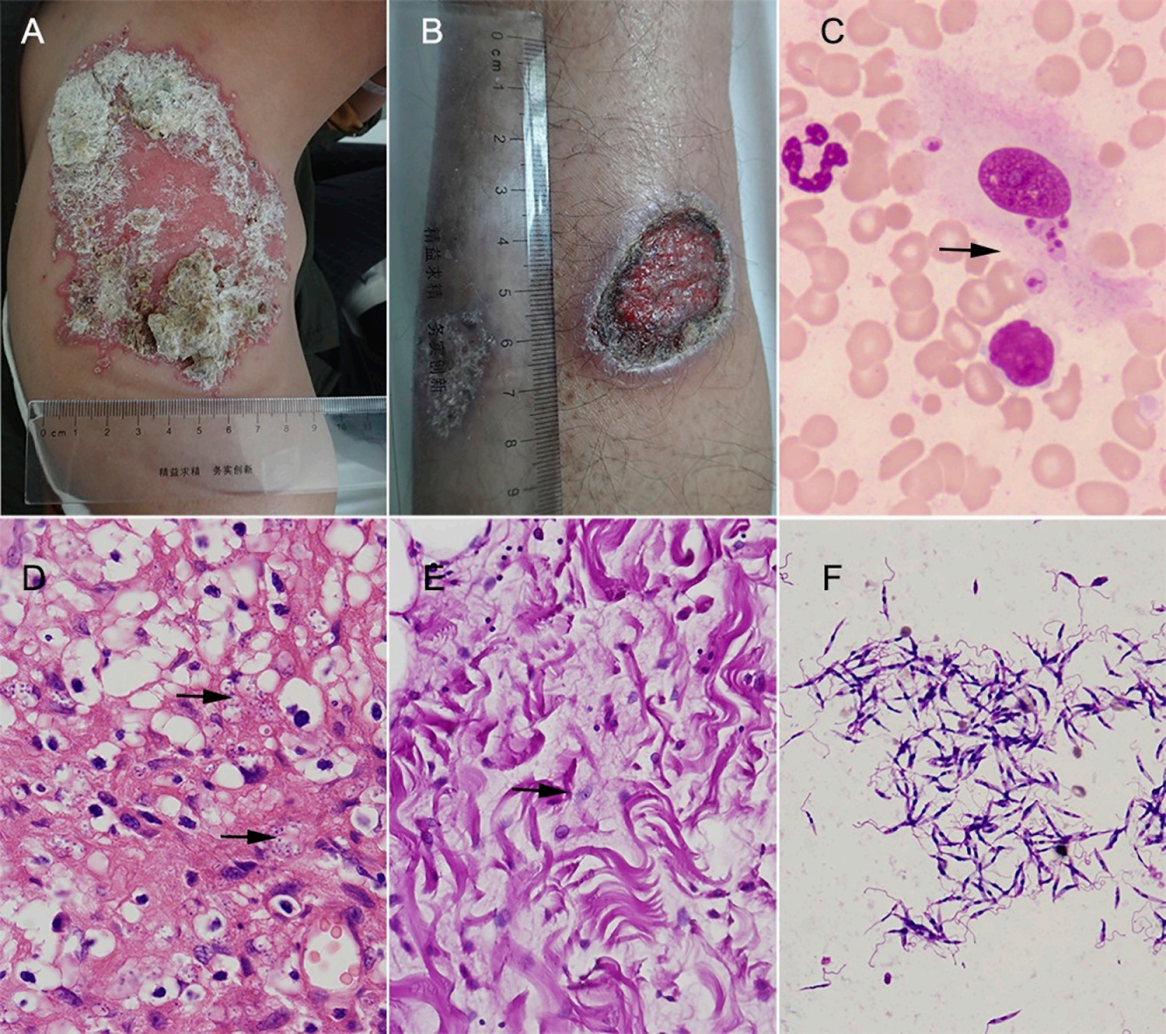
Dermatological, pathological and etiological features of CL skin lesion. **A.** The representative picture of nodular lesion (dry type). **B.** ulcerative lesion (wet type). **C.** *Leishmania* amastigotes detected in lesion smear with Giemsa staining (1000×, arrow). **D-E.** Pathological examination of skin lesion biopsy showing amastigotes (arrowed) stained with H&E (D) or PSA (E) (400×). **F.** *Leishmania* promastigotes detected in lesion scraping culture (Giemsa staining, 1000×).

The clinical characterization of 25 CL cases imported from Iraq are described in Table 3. Most of the cases appeared with skin lesion during the last six month (22/25, 88%), only 3 cases lasted for more than 6 months, indicating they received infection during their work dispatched to Iraq. The skin lesion happened all over the exposed body including limbs, face and back, with most lesion occurred on the limbs (17/25, 68%). The size of most lesion was less than 1 cm (32, 60.3%) with only two cases over 5 cm in diameter. Most of the CL cases have more than one skin lesion (19/25, 76%). There was more ulcerative (wet type, 14/25) lesion than nodular (dry type, 11/25) in these cases (Table 3 and Fig 3A and 3B).

**Table 3:** Clinical patterns and diagnosis of the imported cutaneous leishmaniasis.

Parameters		N = 25	%
Duration of lesions			
	<3month	6	24
	3–6 month	16	64
	>6 month	3	12
Lesion sites			
	Face	4	16
	Limbs	17	68
	Trunk	4	16
Number of lesions			
	1	6	24
	2	12	48
	3	5	20
	>3	2	8
Diameter of the lesions (cm)			
	cm	Lesion#	%
	<1	32	60.3
	1–3	11	20.7
	3–5	8	15
	>5	2	4
Form of lesions			
	Nodular (Dry type)	11	44
	Ulcerative (Wet type)	14	56
Species determination by PCR			
	*Leishmania major*	23	92
	*Leishmania infantum*	2	8
Protozoa identification by lesions smear			
	Positive	22	88
	Negative	3	12
Protozoa identification by pathological biopsies			
	Positive	15	60
	Negative	10	40
Protozoa identification by cultures			
	Positive	13	52
	Negative	12	48

All 25 cases of CL were confirmed by the detection of *Leishmania* parasite either through microscopy of lesion smear and tissues biopsies, or lesion culture with positive promastigotes or PCR amplifications of *Leishmania*-specific ITS-1. In this study, the parasite could be identified in most cases by lesions smear (22/25, 88%) (Table 3 and Fig 3C) and pathological biopsies (15/25, 60%) (Table 3 and Fig 3D and 3E) whereas there was lower rate for the identification of promastigotes in lesion sample culture (13/25, 52%) (Table 3 and Fig 3F). In contrast, PCR amplification of *Leishmania*-specific ITS-1 showed the highest detection rate (25/25, 100%) (Table 3). Based on the sequences of amplified ITS-1, the species of infected parasite was identified mostly as *L*. *major* (n = 23) and less *L*. *infantum* (n = 2).

### 3.4. T Cell phenotypes infiltrated in cutaneous lesions

**Fig 4:**
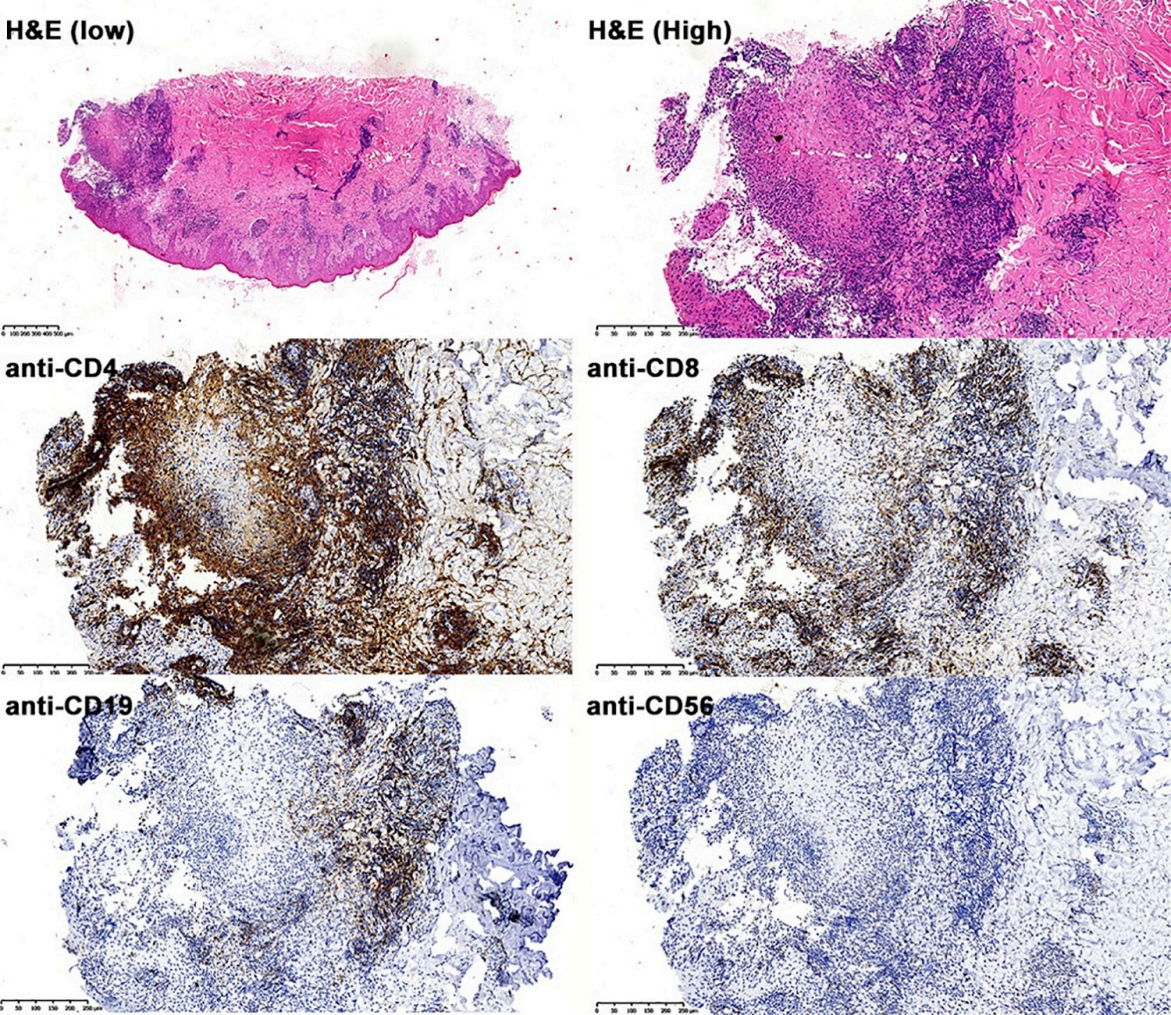
The representative immunohistological staining of immune cells infiltrated in skin lesions. Sections were stained with H&E, anti-CD4, anti-CD8. Anti-CD19 and anti-CD56, respectively (×200).

Immunophenotype of the lymphoid cells infiltrated in the cutaneous lesions can provide information for the cellular immune response in situ during the infection. Based on the immune histopathological staining on fifteen skin lesion biopsy samples, it was showed that the staining area of CD4^+^, CD8^+^, CD19^+^, and CD56^+^ T cells accounted for lesion observed area was 64.8 ± 13.6% (average ± SD), 48.6 ± 9.6%, 21.4 ± 2.2% and 10.7 ± 0.8%, respectively. The results indicated that the dominant T-cells infiltration in the skin lesions were CD4^+^ and CD8^+^ T cells, with less CD19^+^ cells and CD56^+^ cells, indicating *Leishmania* infection in skin lesion mainly induce T-cell response including CD4^+^ help T-cells or cytotoxic CD8^+^ T-cells with less CD19^+^ B-cell response or CD56^+^ NK cells (Fig 4).

### 3.5. Intralesional expression of cytokines profile

**Fig 5:**
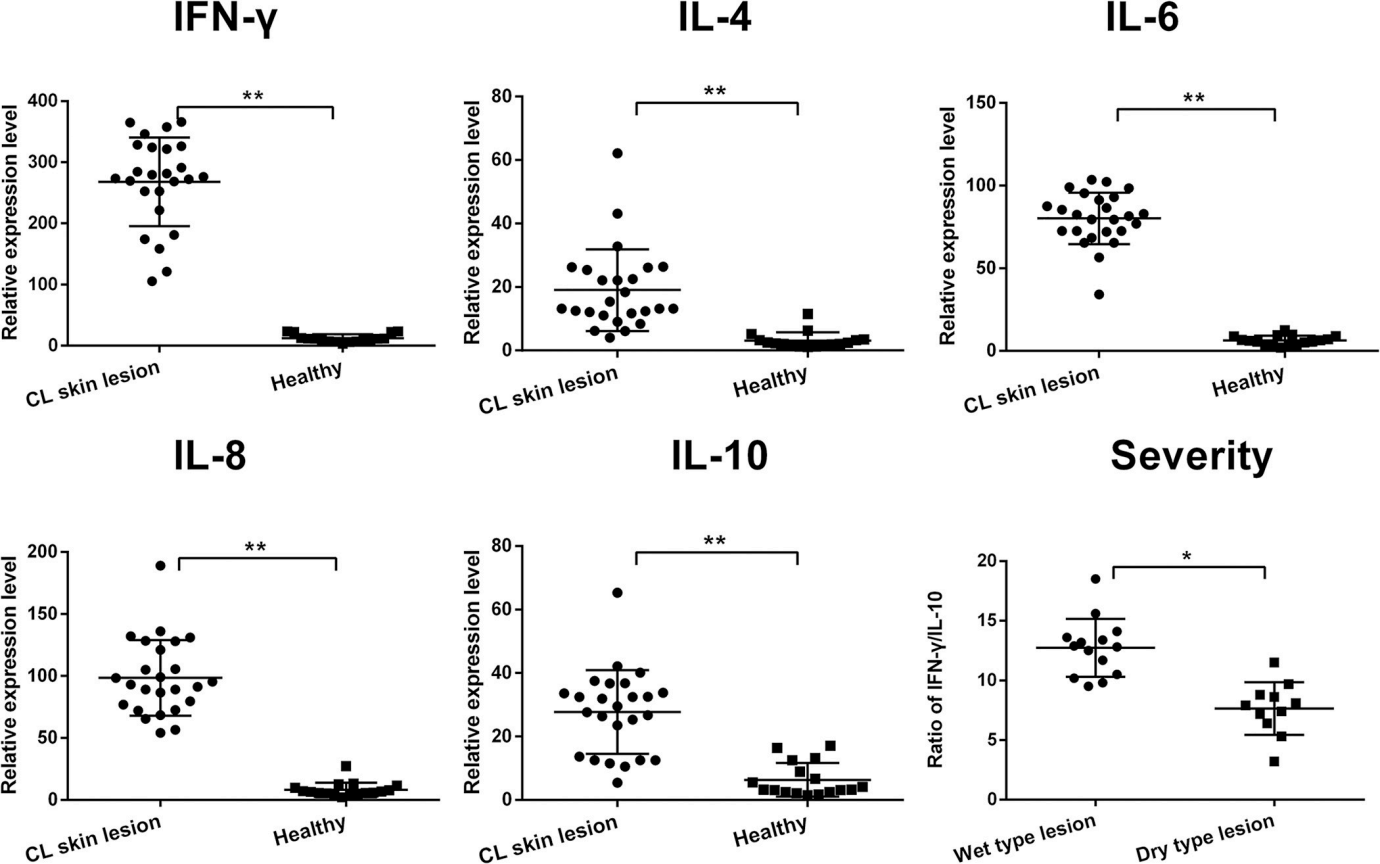
Different cytokine mRNA expression in lesions measured by RT-qPCR. The transcriptional levels of Th1-associated cytokines IFN-γ, IL-6 and IL-8) and Th2-associated cytokines (IL-4 and IL-10) were measured in each lesion biopsy tissue compared to those expressed in healthy skin tissue. The ratio of IFN-γ/IL-10 in wet lesion and dry lesion of CL was shown on the down right. (**P<0.001; *P<0.05).

The transcriptional levels of different cytokines in skin lesions collected from each case were measured by RT-qPCR. Both Th1-associated cytokines, including IFN-γ, IL-6 and IL-8, and Th2-associated cytokines IL-4 and IL-10, were induced in the most of lesions compared to the normal skin tissue without infection. Furthermore, the expression of Th1-associated cytokines was significantly higher than Th2 cytokines in skin lesion (Fig 5). It was reported that the severity of the cutaneous leishmaniasis was related to high level of IFN-γ and low level of IL-10 [31]. We measured the ratio of IFN-γ to IL-10 in each lesion and found that indeed wet lesions (acute in progress) had higher ratio of IFN-γ/IL-10 than that of dry lesions (healing) (Fig 5).

### 3.6. Phylogenetic analysis based on ITS-1 sequences

**Fig 6:**
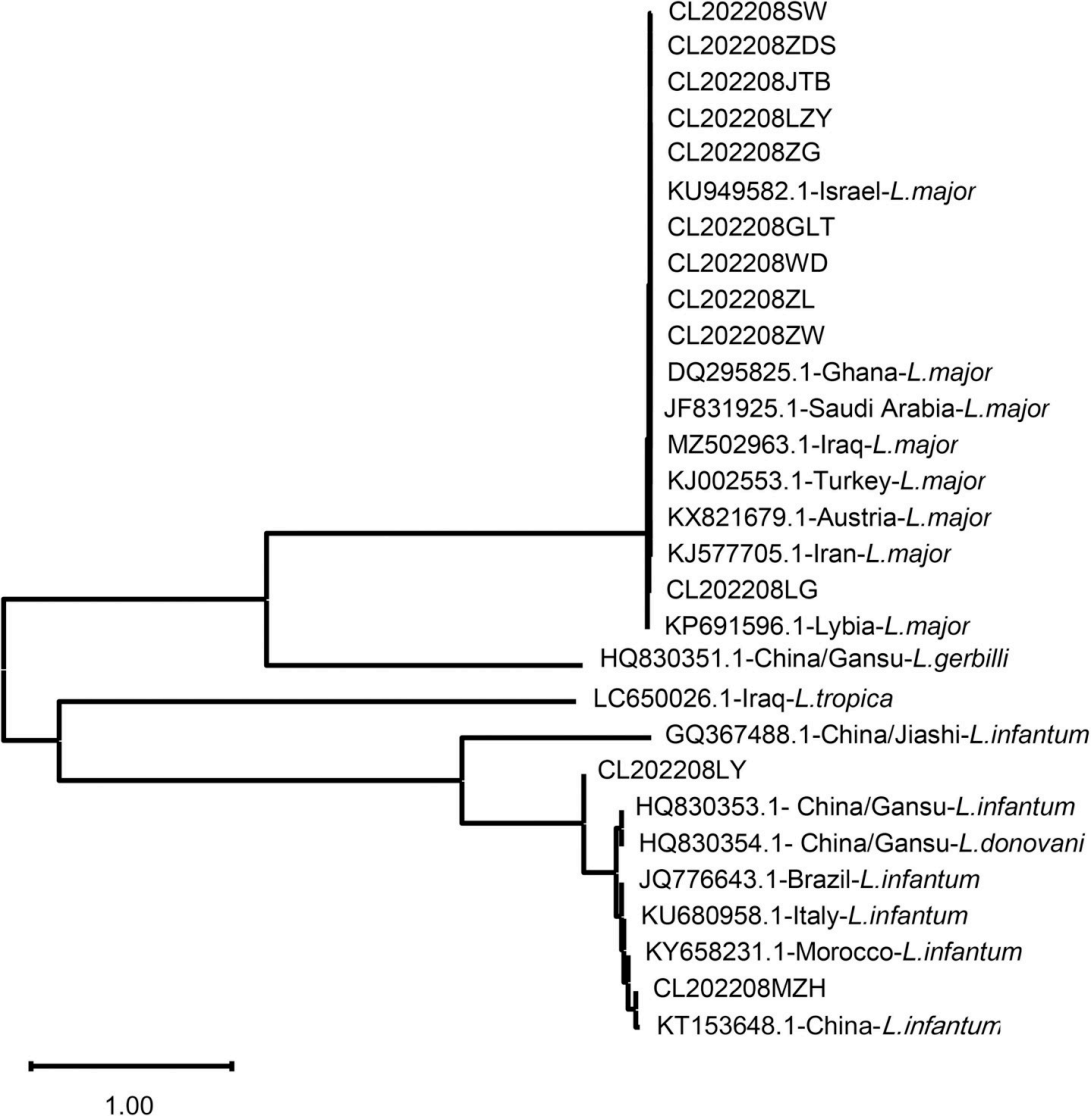
ITS1-based phylogenetic analysis of *Leishmania spp*. isolated from cases returned from Iraq. The phylogenetic tree was constructed based on the ITS1 sequences isolated from 12 cases of Chinese workers infected in Iraq (CL202208SW, CL202208ZDS, CL202208JTB, CL202208LZY, CL202208ZG, CL202208GLT, CL202208WD, CL202208ZL, CL202208ZW, CL202208LG, CL202208LY and CL202208MZH) compared with those known ITS1 sequences deposited in GenBank (GenBank#-region-species) using Neighbor-joining Method. The evolutionary distances were computed using phylogenetic UPGMA tree type (MEGA 11.0 version). The bar at the bottom provides the scale of these branch lengths.

ITS-1 gene was amplified from 12 CL case samples and sequenced. The obtained ITS-1 sequences were aligned with those deposited in GenBank with known *Leishmania* species to construct a phylogenic tree using neighbor-joining method (Fig 6). As shown in the tree, 10 of 12 ITS-1 sequences obtained from the imported CL cases closely related to the isolates from Middle East, indicating their origin in Iraq to which the cases visited and worked. Other two samples were identified as *L*. *infantum* more related to *L*. *infantum* isolates from China or from Mediterranean region or Brazil.

### 3.7. Outcome of treatment

After being treated with intralesional injection of 1–3 mL SSG daily for 6 days, all patients have been recovered with lesion healed without relapse. All patients were tolerant to the intralesional treatment with SSG without apparent side effect.

## 4. Discussion

Cutaneous leishmaniasis is one of the major neglected tropical diseases that threat millions people in Iraq. The poverty, war conflict and climate changes exacerbate the prevalence and morbidity of cutaneous leishmaniasis. The total incidence rate of cutaneous leishmaniasis in Iraq varies from 2.3 to 45.5 / 100000, with estimated 1.0 to 18.0 thousand new infections annually [32].

In China, CL has been only previously reported in Karamay of Xinjiang, west of China [14]. Nowadays, along with the rapid development of the economy of China and high level international investment, more and more Chinese workers or engineers are dispatched abroad annually, especially to those developing countries with endemic of neglected tropical diseases and other infectious diseases. As the consequence, more infectious diseases that are not endemic in China are imported as those migrant workers returned to China [33]. More than 20 thousand Chinese workers are dispatched in Iraq in which they are threatened by the infection of *Leishmania spp*.. Indeed, more and more imported cases of leishmaniasis were reported in China in recent years [21,22,34–39], even though the transmission of the imported leishmaniasis in China was assessed as low risk [36]. However, the clinical and laboratory features of imported leishmaniasis was not well characterized. In this study, we investigated 72 suspected CL cases returned from Iraq and found 33 cases with confirmed CL. Their clinical and parasitic characterization was analyzed and summarized for the first time in order to better understand CL from workers returned from counties where leishmaniasis is endemic.

Analysis of imported CL cases suggested people involved with outdoor work such as construction workers, especially with companion of pet dog in their daily life, had higher chance to get infected. Even though the major reservoir animals for *L*. *major*-caused cutaneous leishmaniasis in Middle East are rodents (jird and rat) based on the WHO Technical Report Series 949 for the Control of the Leishmaniasis [40], dog can not be excluded as the potential zoonotic reservoir animals for *L*. *major* infections [41]. However, the reported cases also observed many rats running around in their living areas. The results in this study also suggest that outdoor activities have more chance to expose themselves to the bite of *Phlebotomine* sandflies carrying the infective promastigotes of *Leishmania spp*., therefore have more chance to get infected. Even though the higher usage rate of insect repellent was observed in the infected people than people without infection that is controversial to the conclusion that the regular use of repellent insecticides is beneficial for the prevention of insect-transmitted leishmaniasis [42]. The reason for this result is possibly because of the insufficient number of observed infected patients and uninfected people and the unknown effectiveness of repellent on the reducing sandfly bites. Cutaneous leishmaniasisis highly prevalent in the eastern and northeastern region of Iraq with highest prevalence in provinces of Sulaymaniyah, Dahuk, and Arbil [43]. The imported CL cases mostly came from the same highly endemic region, but with highest source originated from Maysan and Basrah, the region with most Chinese construction workers.

Ulcerated skin lesion is the major clinical manifestation of CL in Iraq with wet-type lesion more than nodule dry-type lesion (63.5% vs 36.5%). It indicates the zoonotic cutaneous leishmaniasis caused by the *L*. *major* (wet-type) is more than the anthroponotic cutaneous leishmaniasis caused by the infection of anthroponotic *Leishmania spp*. (dry-type) in Iraq/Iran areas [44]. The high incidence of ulcerative wet type lesions indicates higher infection and transmitted from the reservoir animals in the endemic region especially canid sand rodents [32]. In this study, we identified 56% (14/25) of Chinese CL cases (14/25) with ulcerative wet lesion and 44% (11/25) with nodular dry lesion that is similar to the ratio of local Iraqi CL patients. In addition, *L*. *major* is the major species that causes ulcerated lesions in Iraq [45], it is consistent with the species identified in Chinese cases (92% (23/25) with *L*. *major*.

The non-specific skin lesion of cutaneous leishmaniasis could be misdiagnosed as other dermatoses, resulting in inappropriate treatment and unnecessary delay of healing. Thus, the parasite identification is necessary for the definite diagnosis of *Leishmania* infection, especially for those cases with travel history in endemic areas. In this study, a comprehensive diagnostic algorithm was applied for the diagnosis of CL for those returned from a travel to endemic Iraq including protozoa identifications directly from lesion smear or culture in vitro, skin biopsy pathology, PCR amplifications of *Leishmania*-specific ITS-1. As a result, PCR analysis is currently the most sensitive method for detection of *Leishmania*. The sequence ITS-1 was amplified from all CL cases that can be used as the golden standard for the *Leishmania* infection, it also can be used to determine the species of infected *Leishmania* parasite [46,47]. In this study, the sequence alignment of ITS-1 amplified from CL cases determines that 23 out of 25 confirmed CL cases were infected with *L*. *major*, that is consistent with the major species endemic in Iraq [45]. However, we found 2 cases infected with *L*. *infantum* that causes infantile visceral leishmaniasis in the Mediterranean region [48], also found in the western China [49] that even induced deadly secondary hemophagocytic lymphohistiocytosis [50]. Interesting, there was no *L*. *tropica* detected in the CL cases who returned from Iraq, which is also the endemic species in Iraq with zoonotic cutaneous leishmaniasis (ZCL) [51], possibly because Chinese workers usually lived together in the urban region where the *L*. *major* infection is more dominant than *L*. *tropica*. Quantitative PCR also can be used to determine the parasite load and the therapeutic efficacy during treatment. Except for PCR assay, lesion smear examination is a simple, inexpensive and rapid approach to determine the *Leishmania* infection especially suitable for the local clinic in endemic areas.

It has been reported that T cells play a critical role in the clinical presentation and course of leishmaniasis. The immnuohistopathological evaluation of skin lesion tissues revealed that the infected skin tissue was highly infiltrated by T cells, with predominance of CD4^+^ T cells over CD8^+^ T cells. Conversely, much less CD19^+^ (B cell linage) or CD56^+^ (natural killer cell) cells were observed in the lesions, indicating that mainly T cell-mediated immune-response, rather than humoral immunity or innate immunity, has been involved in the skin tissue lesion and disease progression [52]. The cytokine profile measured in lesion tissue is consistent with the dominant T cell responses with higher inflammatory cytokines IFN-γ, IL-6 and IL-8 and less anti-inflammatory cytokines including IL-4 and IL-10 [53]. IFN-γ mediated Th1 immune response is not only involved in the pathology of CL lesion but also has been reported to protect against sand fly delivered *Leishmania* infection [54,55].

## 5. Conclusion

In this study, we have identified 25 CL cases in migrant Chinese workers returned from Iraq with *L*. *major* as the major species of infected *Leishmania* parasite. Clinical features of the Iraq-imported CL include the history of skin exposure to sandflies bite and the lesions mostly on the exposed limbs. More ulcerative wet lesion was observed than nodular dry lesion. The definitive diagnosis of CL is based on the parasite identification by microscopic examination directly on lesion smear or parasite culture, PCR amplification of *Leishmania*-specific ITS-1. PCR is not only used to detect *Leishmania* parasite with high sensitivity, but also to identify the species of infected parasite through sequencing the amplified ITS-1 gene. The phylogenetic analysis based on the amplified ITS-1 sequences revealed that the infected *Leishmania* was closed related to the species and strains endemic in Iraq. The immunopathological examination revealed the T-cell filtrated cellular immune response with less B cells and NK cells involved. The cytokine profile measured in the skin lesion also confirmed the Th1 cellular response with higher expression levels of IFN-γ, IL-6 and IL-8. The lesions were sensitive to the treatment of antimony locally and all patients were tolerant to the intralesional injection of antimony without apparent side effect. The clinical and parasitological features of these Chinese CL cases imported from Iraq provide useful information for the diagnosis and treatment of CL that is not commonly seen in Chinese local population.
